# A missense mutation in the *MACF1* gene in a patient with autism spectrum disorder and epilepsy

**DOI:** 10.25122/jml-2024-0312

**Published:** 2024-11

**Authors:** Alexandru Capisizu, Carmen Sandu, Roxana Maria Caragea, Adriana Sorina Capisizu

**Affiliations:** 1 Dr. Constantin Gorgos Psychiatry Hospital, Bucharest, Romania; 2 Department of PhD studies, Division of Physiology and Neuroscience, Department of Functional Studies, Carol Davila University of Medicine and Pharmacy, Bucharest, Romania; 3 Department of Pediatric Neurology, Dr. Alexandru Obregia Psychiatry Hospital, Bucharest, Romania; 4 Department of Radiology and Imagistic Medicine 1, Faculty of Medicine, Carol Davila University of Medicine and Pharmacy, Bucharest, Romania

**Keywords:** autism spectrum disorder, case report, *MACF1*, epilepsy

## Abstract

The *MACF1* gene (OMIM: 608271) encodes the Microtubule-Actin Cross-Linking Factor 1 protein. Existing medical research shows that genetic mutations in the *MACF1* gene have been associated with neurodevelopmental and neurodegenerative disorders, with variants of unknown significance also linked to autism spectrum disorder (ASD). However, the number of reported autism disorder or epilepsy cases associated with *MACF1* mutations remains limited. We present the case of a 7-year-old girl, a long-term patient at the Pediatric Neurology Clinic of Dr. Alexandru Obregia Hospital in Bucharest, followed since the age of 3. She initially presented with epilepsy characterized by generalized seizures, clinically resembling both spasms and myoclonus. Over time, she exhibited features of a pervasive developmental disorder and moderate cognitive delay. Genetic testing identified a missense point mutation in the *MACF1* gene, c.16223C > T, p.(Pro504Leu). Her final diagnosis was epilepsy with generalized seizures of non-lesional origin, moderate cognitive impairment, pervasive developmental disorder, and a confirmed point mutation in the *MACF1* gene. This case underscores the importance of incorporating genetic testing into the diagnostic process for patients with autism spectrum disorder and epilepsy.

## INTRODUCTION

The *MACF1* gene (OMIM: 608271), a gene located on the short arm of chromosome 1, encodes the protein Microtubule-Actin Cross-Linking Factor 1, also known as Actin Cross-Linking Factor 7, which is essential for the proper modulation of the cytoskeletal network, made up of actin and microtubules [1-6]. Existing medical research shows that genetic mutations in the *MACF1* gene have been associated with defects in neuronal migration and axonal function, neurodevelopmental and neurodegenerative disorders, being frequently associated with schizophrenia, Parkinson's disease, and with cases of Lissencephaly and complex malformations of brain stem [7-12]. Furthermore, variants of unknown significance (VUS) in this gene have also been associated with autism spectrum disorder (ASD) [7].

### Patient information and history

The patient is the third child of a healthy, non-consanguineous couple. In the family, there is no history of neurodevelopmental disorders, epilepsy, or known neuromuscular or genetic diseases. The pregnancy was classified as high-risk due to placenta previa and metrorrhagia. Delivery occurred via cesarean section at 34 weeks, with an Apgar score of 8 and a birth weight of 2400g. As a postnatal adaptation, she spent five weeks in the incubator and received antibiotic therapy. She was discharged from the maternity ward after two weeks, healthy, and vaccinated. She walked independently at the age of 10 months. The first syllables appeared at 6 months, and she began to speak words at the age of 1 year. At the age of 2 years and 3 months, epileptic seizures began, making seizure control challenging. Over time, behavioral disturbances became evident, including oppositionality, hyperactivity, and delayed cognitive development, particularly in language.

### Clinical findings

At 7 years and 3 months, the patient shows normal nutritional development, with a weight of 25 kg and a height of 126 cm. Her cranial perimeter measures at the 90th percentile. She presents some particular facial features: epicanthus and hypertelorism. No other constitutional features have been identified. No skin abnormalities have been identified, such as rashes or cafe-au-lait spots. Cardiopulmonary clinical assessments are within normal limits.

Neurological evaluation revealed no deficits, no signs of meningeal irritation, and no abnormalities in examining the cranial nerves or motility. There was no segmental motor deficit, muscle tone or strength changes, or coordination deficit. There were no abnormalities of the osteo-tendinous reflexes, and there was no Babinski sign. Also, no abnormalities of sensitivity have been identified.

### Timeline

**Figure 1:**
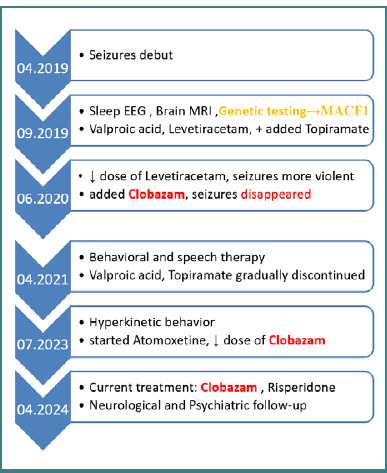
Clinical evolution and treatment over the years

Starting from a psycho-motor development and evolution within normal limits until the onset of epileptic seizures, the information captured in Figure 1 focuses on the subsequent clinical and therapeutic evolution, emphasizing the difficulties in obtaining control over epileptic seizures and marking the gradual appearance of complex psychiatric symptomatology.

At the age of 2 years and 3 months, the paroxysmal manifestations debuted, with the extension of the upper limbs, which appeared in clusters, lasting one or two seconds, some of them related to falling asleep, others unrelated to sleep, initially rare, up to 3 episodes per day.

**Figure 2:**
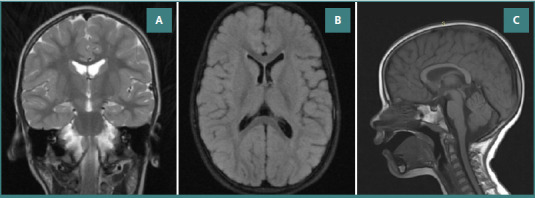
MRI examination. A, Coronal T2 examination with a normal aspect of the cerebral hemisphere, cerebral ventricles, and brainstem. B, Axial FLAIR image without anomalies of white cerebral matter, basal ganglia, and cerebral cortex. C, Sagittal T1 examination without signal or morphological abnormalities in the cerebral hemispheres, brain stem, pericerebral fluid spaces, basal cisterns with morphology within normal limits, and cerebellar tonsils normally located.

By 2 years and 8 months, during her first consultation at the Pediatric Neurology Clinic, a first long-term wake and sleep video-electroencephalography (EEG) investigation showed epileptic discharges of polyspikes and waves, as well as slow spike-wave complexes during wakefulness. The sleep recording showed the persistence of these discharges described in the awake stage, more frequent than during wakefulness. During recording, the girl only presented a subtle startle, with abduction of the upper limbs and a poly-spike wave-type discharge simultaneously on the EEG trace. At the same age, a brain MRI scan showed a normal aspect of the cerebral hemispheres, cerebral ventricles, and brainstem, slightly dilated Virchow spaces, and no etiologically relevant lesions (Figure 2A-C).

Genetic testing using the Blueprint Genetic’s 'Beyond Pediatric Epilepsy PLUS' panel revealed a missense mutation in the *MACF1* gene, classified as a variant of unknown significance (VUS). Additionally, a Portage test for psychomotor development was performed, which showed a slight delay in language and cognitive development.

During this admission, the decision was made to introduce anti-epileptic treatment, initially with Valproic acid, gradually increased to an appropriate dose, up to 30 mg/kg/day, without significant improvement in seizure frequency. Thus, Levetiracetam was added and gradually increased to an appropriate dose of 50 mg/kg/day. In the following weeks following discharge from the hospital, the seizures were not pharmacologically controlled. The frequency of seizures remained constant, without improvement: 4-6 clusters a day, with the highest cluster of 26 seizures.

At **2 years and 9 months**, at her subsequent follow-up, Topiramate was introduced as a third anti-epileptic drug and slowly increased to 6.7 mg/kg/day. The follow-up EEG showed no improvement.

At **3 years and 1 month**, after reducing the dose of Levetiracetam, the manifestations became more sudden and more pronounced; therefore, as the EEG epileptiform anomalies were maintained, treatment with Levetiracetam was not interrupted.

At **3 years and 5 months**, at her subsequent clinical evaluation, as the seizures continued more violently since the Levetiracetam dose was reduced, a decision was made to add a benzodiazepine to the therapeutic plan, thus choosing Clobazam. After the introduction of Clobazam, the seizures disappeared. Also, Levetiracetam was gradually discontinued. In addition, during this visit, concerns regarding her neuropsychiatric development were identified. The patient displayed signs of inattention, hyperactivity, and difficulty cooperating during the examination. She demonstrated impatience with activities, oppositional behavior, and poor language development, indicating significant challenges in communication and overall cognitive progress.

At **3 years and 8 months**, at her periodic follow-up, the girl was seizure-free. The neurological examination showed a delay in the development of expressive language and some behavioral features. The Portage test, performed again this time, showed a developmental quotient (DQ) level 75. She was at the time consulted by a pediatric psychiatrist, who recommended the initiation of behavioral therapy sessions aimed at her hyperkinetic behavior. Regarding the anti-epileptic treatment, Valproic acid was gradually discontinued while continuing treatment with Topiramate, 6 mg/kg/day, and Clobazam, 0.81 mg/kg/day. The wake and sleep EEG showed an improved course without epileptiform discharges.

At **4 years and 3 months**, still seizure-free, speech therapy was also recommended. Her wake and sleep EEG showed a similar path to the previous recording, clearly improved, without epileptiform changes, so it was recommended to continue the treatment with Clobazam and gradually decrease the dose of Topiramate.

At **4 years and 8 months**, after behavioral and speech therapy sessions, 10 hours/week, she was making progress, being much calmer, and respecting the rules in therapy. Also, new progress in language was observed as she started forming sentences. As the EEG pathway was improved, the treatment plan remained similar, with both Clobazam and Topiramate, the latter being gradually decreased.

At **5 years and 4 months**, improvement in behavior and psycho-visual contact was observed: she was forming sentences and understanding simple commands, social interaction was present, and she repeated words upon persuasion. Oppositionalism and hyperkinesia were noted, and dyslalia was still present. At that time, Topiramate treatment was permanently discontinued, and she continued with Clobazam, 0.62 mg/kg/day. EEG findings revealed occasional bursts of sharp waves during sleep without clinical correlation.

At **6 years and 6 months**, clinical assessment mentioned cognitive progress, but hyperkinetic behavior and the lack of concentration were maintained. The recommendation of the pediatric psychiatrist was the initiation of drug treatment for hyperkinetic syndrome, Atomoxetine. The wake and sleep EEGs performed in this period showed isolated elements of bilateral frontal spike-and-wave complexes and unsystematized spikes. Clobazam treatment was continued with a decreased dose.

**Figure 3:**
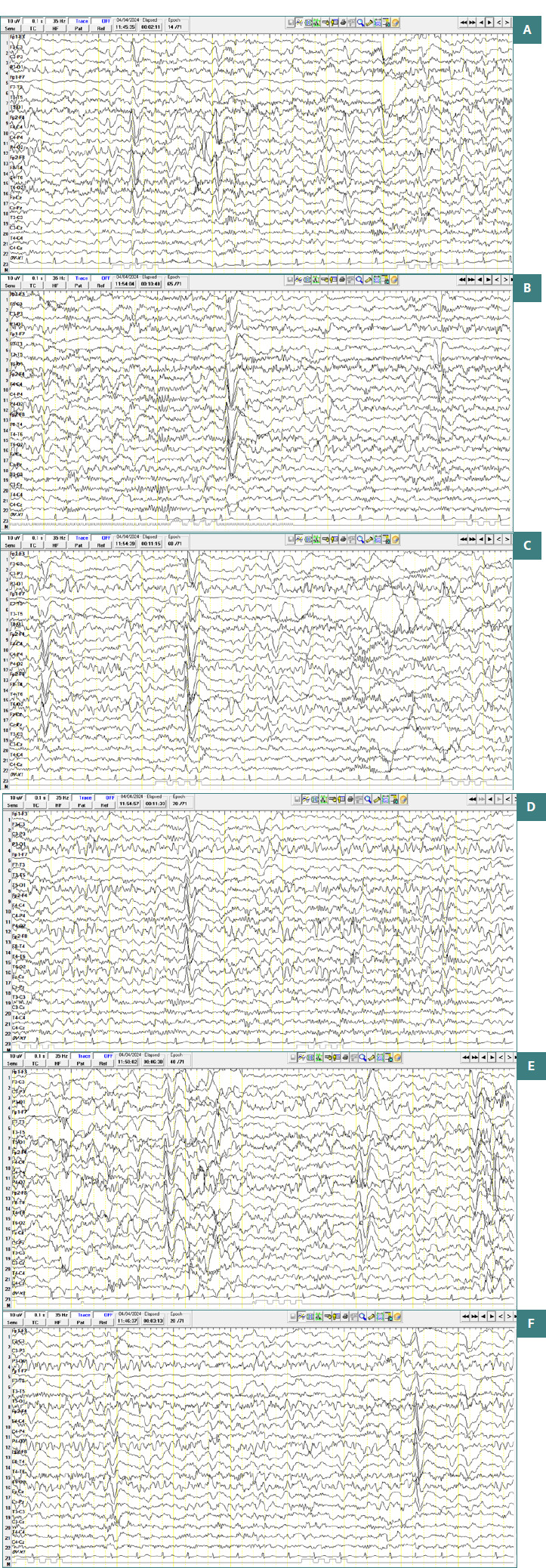
Wake EEG recordings show symmetrical background activity with spike discharges and spike-and-wave complexes localized bilaterally in the frontal regions, more frequent on the right side compared to the left. These discharges have an amplitude of approximately 150 µV and are observed consistently throughout the recording in both eyes-open and eyes-closed conditions. Activation tests, including hyperventilation (HV) and intermittent photic stimulation, did not induce any additional pathological changes. A, Spike-type discharges and spike-and-wave complexes with bilateral frontal localization in the eyes open recording; B, eyes closed recording; C, D, E, Recordings during hyperventilation; F, Eyes-closed recording following hyperventilation.

At her last check-up, at **7 years and 3 months**, the girl presented episodes of agitation and oppositionalism and cooperated with difficulty during the examination. In terms of language, she has shown some good development. It was recommended that she continue cognitive-behavioral therapy sessions. Also, Atomoxetine treatment was gradually replaced with Risperidone because of decreased appetite and weight loss. As she remained seizure-free since the age of 3 years and 8 months, the girl continued treatment with Clobazam, 0.4 mg/kg/day, and her wake EEG showed spike-type discharges and spike-and-wave complexes with bilateral frontal localization (Figure 3A-F).

### Evolution and perspective

The patient is currently on anti-epileptic treatment with Clobazam at a dose of 0.4 mg/kg/day and has been seizure-free since the age of 3. In addition to pharmacological management, she is actively engaged in cognitive-behavioral therapy sessions aimed at addressing her hyperkinetic behavior and developmental delays. Since the onset of her seizures at 2 years and 8 months, she has been closely monitored through regular clinical and neurological evaluations, including sleep EEGs, at the Pediatric Neurology Clinic of Dr. Alexandru Obregia Hospital in Bucharest. Her follow-up schedule is maintained at three-month intervals, with additional visits planned as necessary, particularly in the event of any clinical deterioration. Following the emergence of psychiatric symptoms, she has also been under the care of the Pediatric Psychiatry Clinic at Dr. Constantin Gorgos Hospital in Bucharest. Regular psychiatric evaluations are conducted every three months to assess her progress and adjust her therapeutic regimen as required.

## DISCUSSION

In this article, we present the case of a 7-year-old girl diagnosed with a rare point missense mutation in the *MACF1* gene, c.16223C > T, p. (Pro504Leu), classified as a variant of uncertain significance. As this specific VUS has not previously been reported, we can only judge the significance of this mutation, on the one hand, through the reports in the available literature, those of previous cases of *MACF1* gene mutations discovered, and, on the other hand, we can share the conclusions related to this VUS, about how our patient was affected, in terms of both development and response to treatment.

Until 2009, Sanchez-Soriano and colleagues indicated that *MACF1* played a vital role in neurite outgrowth [6], and in the last 10 years, researchers presented the link between *MACF1* and the nervous system and its disorders in both children and adults. In 2018, Moffat *et al*. highlighted the role of *MACF1* in proliferation, migration, and neurite outgrowth in neurons and neural progenitors [7]. Brain malformations related to *MACF1* mutations have been described by Dobyns *et al*. in 2018 [8]. They first recognized a rare lissencephaly variant with a complex brainstem malformation with pontine clefts, wide and flat medulla, and visible pyramids on the ventral surface in unrelated children. All eight children had severe developmental delay, spasticity, and seizures within the first year of life. These clinical and imaging features were considered pathognomonic by the authors and by Bölsterli *et al*. in 2020, further supporting the specific association of these anomalies with *MACF1* mutations. Genetic testing revealed de novo missense variants or an in-frame deletion involving the GAR domain of *MACF1* in the eight subjects [9].

In 2020, Bölsterli *et al*. reported a pathogenic de novo *MACF1* variant [c.15682G>T p.(Asp5228Tyr)], during a review of an unsolved brainstem malformation, in a girl who had treatment-resistant epilepsy, spastic cerebral palsy and severe speech and cognitive impairment [9]. They concluded that it resembled the series of previously described cases by Dobyns *et al*. in 2018 [8].

Several psychiatric and neurodevelopmental disorders have been linked to *MACF1* mutations. In 2007, Camargo and colleagues identified *MACF1* as a protein interacting with two schizophrenia risk genes, *DISC1* (disrupted in schizophrenia 1) and *DTNBP1* (dysbindin) [10]. A 2018 study by Moffat *et al*. showed a link between genetic mutation or dysregulation of the *MACF1* gene and neurodevelopmental and neurodegenerative diseases, specifically schizophrenia, ASD, and Parkinson’s disease [7]. Most recently, a 2023 review by Salem *et al*. highlighted the functions of *MACF1* in the nervous system, particularly in the pathogenesis of bipolar affective disorder, as well as in other psychiatric disorders [11-14].

Similar to cases reported in the literature, our patient presents with epilepsy; however, unlike the cases described by Dobyns *et al*., she did not exhibit cerebral malformations on brain imaging [8]. Moreover, she had no signs of severe intellectual disability. Numerous associations between epilepsy and autism have been linked to numerous types of gene mutations, such as genetic syndromes (like maternally inherited chromosome 15q11-q13 syndrome, Down syndrome, Phelan-McDermid syndrome) or single gene disorders (like Fragile X Syndrome, Tuberous Sclerosis Complex, *MECP-2*-related syndrome) [15,16]. Fragile X Syndrome, for instance, is associated with facial features, intellectual disability, and epilepsy in 10-20% of all cases [17]. In contrast, our patient with a *MACF1* mutation did not demonstrate comparable psychiatric impairments, and the EEG discharges are different from those found in Fragile X Syndrome.

The Tuberous Sclerosis Complex, caused by mutations in the *TSC1* or *TCS2* genes, results in abnormalities in cell growth and differentiation through disruption of the mTOR (mammalian target of rapamycin) pathway. Compared to our case, the epileptic impairment in the Tuberous Sclerosis Complex is largely focal and is due to significant cerebral lesions, mainly the cortical tubers. Also, the Tuberous Sclerosis Complex produces greater cognitive and behavioral impairment [18].

Also, regarding *MECP2* gene mutations and the Rett phenotype, the common existence of expressive language disorder and epileptic seizures is noted, but the characteristic midline movement abnormalities, or its specific facial dysmorphism, are missing [15].

Regarding anti-epileptic treatment, during the medical checks in the pediatric neurology clinic, up to four broad-spectrum anti-epileptic treatments were gradually introduced, in monotherapy and then pluritherapy, but these had to be stopped one by one due to their inefficiency.

Clobazam, a benzodiazepine, proved to be the most effective treatment, successfully controlling the patient’s seizures and ultimately serving as the sole agent in monotherapy. Its efficacy across a broad spectrum of epilepsy types—including focal-onset seizures, epileptic spasms, and generalized-onset seizures—was demonstrated in this case, particularly for managing myoclonic-like seizures [19].

Consistent with other published reports, in particular Salem *et al*. [11], the patient associates a psychiatric disorder, in her case, a specific combination of pervasive disorder, hyperkinetic disorder with attention deficit, and oppositional behavior. Unlike the findings of Salem *et al*., our patient did not present severe psychiatric disorders, such as schizophrenia, bipolar disorder, or schizoaffective disorder. In contrast to the report of Forstner *et al*. [13], there was no family history of these medical conditions. It should also be noted that there is an absence of other signs or symptoms associated with the *MACF1* mutation, such as parkinsonism or neuromuscular deficit, as found by Moffat *et al*. [7].

## CONCLUSION

In conclusion, identifying a mutation in the *MACF1* gene necessitates a comprehensive management approach that prioritizes monitoring the patient's developmental milestones, conducting detailed neurological examinations, and screening EEGs. Special attention should also be given to any clinical manifestations, with long-term EEG monitoring employed for suspected epileptic activity. Moreover, in managing cases of autism spectrum disorder associated with epilepsy, genetic testing should play a central role in guiding diagnosis and treatment strategies. In prospective, further research can explore the link between *MACF1* gene mutations and epileptogenesis.

## Data Availability

Further data is available from the corresponding author upon reasonable request.
